# A Novel Defect Estimation Approach in Wind Turbine Blades Based on Phase Velocity Variation of Ultrasonic Guided Waves

**DOI:** 10.3390/s21144879

**Published:** 2021-07-17

**Authors:** Renaldas Raišutis, Kumar Anubhav Tiwari, Egidijus Žukauskas, Olgirdas Tumšys, Lina Draudvilienė

**Affiliations:** 1 Ultrasound Research Institute, Kaunas University of Technology, K. Baršausko St. 59, LT-51423 Kaunas, Lithuania; k.tiwari@ktu.lt (K.A.T.); e.zukauskas@ktu.lt (E.Ž.); olgirdas.tumsys@ktu.lt (O.T.); lina.draudviliene@ktu.lt (L.D.); 2 Department of Electrical Power Systems, Faculty of Electrical and Electronics Engineering, Kaunas University of Technology, Studentų St. 48, LT-51367 Kaunas, Lithuania

**Keywords:** wind turbine blade, finite element model, non-destructive testing, ultrasonic guided waves, defect detection and sizing, phase velocity measurement, macro-fibre composite transducer

## Abstract

The reliability of the wind turbine blade (WTB) evaluation using a new criterion is presented in the work. Variation of the ultrasonic guided waves (UGW) phase velocity is proposed to be used as a new criterion for defect detection. Based on an intermediate value between the maximum and minimum values, the calculation of the phase velocity threshold is used for defect detection, location and sizing. The operation of the proposed technique is verified using simulation and experimental studies. The artificially milled defect having a diameter of 81 mm on the segment of WTB is used for verification of the proposed technique. After the application of the proposed evaluation technique for analysis of the simulated B-scan image, the coordinates of defect edges have been estimated with relative errors of 3.7% and 3%, respectively. The size of the defect was estimated with a relative error of 2.7%. In the case of an experimentally measured B-scan image, the coordinates of defect edges have been estimated with relative errors of 12.5% and 3.9%, respectively. The size of the defect was estimated with a relative error of 10%. The comparative results obtained by modelling and experiment show the suitability of the proposed new criterion to be used for the defect detection tasks solving.

## 1. Introduction

The composite structures such as carbon fibre-reinforced polymers (CFRP) and glass fibre-reinforced polymers (GFRP) are extensively used in aerospace, aeronautic, marine, wind turbines and automotive industries due to their high stiffness to weight ratio, load-carrying capacity, damping properties and much more [1]. Generally, in all applications, where high-performance materials and structures are required, composites are being used. One such application of composites is the wind turbine, and more specifically, the wind turbine blade (WTB) [2]. As WTBs must be sustained in the variable wind or cyclic loads, it is the most defect-prone and sensitive element of the wind turbine. WTB costs around 10 to 20% of the total installation cost of the whole turbine [3]. However, failure of a WLB could lead to failure of the whole turbine; therefore, an overall loss could be much bigger than the cost of a WTB. That is why regular maintenance and inspection of WTBs are necessary to avoid any system failure [4,5].

There are various non-destructive testing (NDT) methods that are used for testing such type of complex structures and analysis of defects [6]. However, the dimension and complexity of WTB and limitation in applicability and accuracy of some methods make them unsuitable for on-site inspection of WTB [7]. Due to the availability of a wide range of transducers, high sensitivity to defects and the ability to travel a long distance, ultrasonic guided wave (UGW) testing is extensively used for the inspection of these type of structures [8,9,10]. The Lamb wave comes under the specific category of UGW that can be further categorized into the two types: symmetric (S0, S1) and asymmetric (A0, A1) Lamb waves based on the product of frequency (*f*) of excitation and thickness (*d*) of the propagating medium. At low frequencies, fewer guided Lamb modes exist, and it becomes easier to perform the mode separation. As a consequence of high sensitivity in the regions of defects, guided Lamb waves are extensively applicable for the inspection of structures containing different kinds of defects such as cracks, holes, delamination, etc. [11,12,13].

Irrespective of all good features associated with UGW, the real-time testing of WTB is still a quite complex and challenging task due to its complex structure, variable thickness and one-sided access. Moreover, the phenomenon and behaviour of UGW in layered composite structures creates more complexity due to various mechanism such as dispersion, reflection, refraction, mode conversion, etc. After considering all these challenges associated with the complex geometry of WTB and the different mechanism of UGWs, the WTB was selected as an object of this research.

Many researchers experimentally reported the defect estimation of composite structures based on the UGW interaction [7,14,15]. Moreover, many studies have been conducted to understand the dispersive and multimodal behaviour of UGWs [16,17,18,19,20]. It should be taken into account that attenuation and scattering of ultrasound are the key challenges to investigate the layered composite structures that can hinder the accurate defect estimation. The attenuation and scattering depend on the wavelength and frequency; that is why low-frequency (LF) ultrasound has been used in this work [21]. Moreover, at LF, fewer guided Lamb modes propagate that could be easier to separate. The low-frequency (LF) guided waves are widely used for the inspection of larger structures such as pipe, rail, WTB, etc. [22,23,24]. In our previous research work, the experimental investigation of WTB using low-frequency UGWs was performed where the variations in energy or amplitudes of the UGW were utilized to locate and size the milled-type defect on the segment of WTB [2,24,25]. However, in the case of UGW, the signal amplitude decreases with distance due to the dispersion phenomenon and spreading of wavefront. Therefore, the usually applied criterion according to the signal amplitude decrease is not appropriable for the defect evaluation in the UGW applications. So, based on that, a new criterion for defect detection and parameterization is necessary to be developed.

The defects in WTB can be generated during the manufacturing process or on-field applications. The shape of the defect depends on various conditions during manufacturing or in usage. During the infusion process, gas holes, delamination or disbond-type defects might appear. Under the comprehensive effect of cyclic load due to wind, vibrations and other factors such as heat, acid and alkali, initial defects could be extended; in turn, the new defects such as fracture, holes, delamination or disbonding can be generated. Defects could also be generated due to random human error such as inadequacy of adhesive glue [26]. The scope of this paper is to explore the alternative approach for the defect analysis in WTB; thus, the simplified hole-type defect is considered in this research.

The objective of this research work is to perform detection, location and sizing of the artificially milled defect having a diameter of 81 mm on the segment of WTB by using the variation in phase velocity of UGW. To our knowledge, in the field of ultrasonic NDT, this is the first time that the defect on WTB has been modelled and its size estimated based on phase velocity variations of UGW.

The paper is organized as follows: Section 2 describes the FE modelling of WTB containing a milled defect. The three-step signal processing: removal of back-reflected waves by using 2D-FFT, the selective extraction of the A0 mode and defect sizing using phase velocity variations are expressed in Section 3. The experimental investigation of WTB using the LF ultrasonic system is presented in Section 4. Finally, the conclusive remarks are summed up in Section 5.

## 2. The Sample of WTB Containing a Drilled Defect and FE Modelling

**Figure 1:**
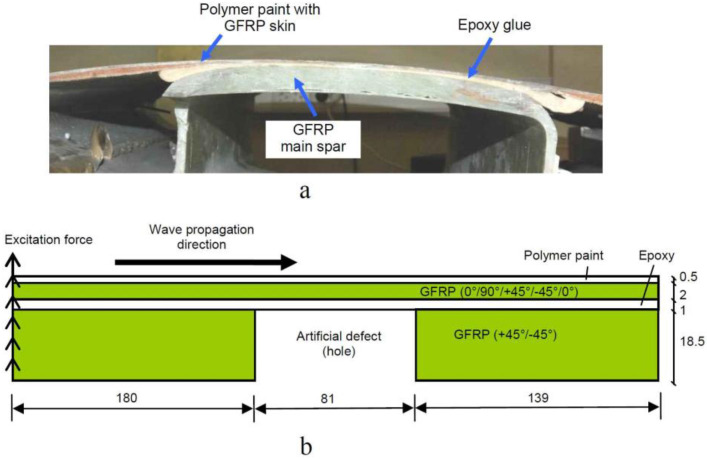
The real WTB sample (**a**) and graphical representation of the structure with a drilled defect (hole) of 81 mm diameter (**b**).

The cross-section image of the sample of WTB is shown in Figure 1a. The most possible place for the appearance of disbond-type defects is between the epoxy adhesive layer and main spar and covering skin. It was not possible to manufacture a disbond-type defect in the real sample. Hence, the hole in the main spar was milled through the whole thickness of the main spar up to the epoxy adhesive layer.

The graphical representation of WTB model under analysis is presented in Figure 1b. The material of the main spar was removed in the defect position for modelling the defect as similar to the real WTB sample available in our lab.

In order to analyse the propagating guided waves in the multi-layered structure of WTB, the dispersion curves were calculated using the semi-analytic finite element (SAFE) technique [27,28,29]. The frequency-dependent attenuation coefficient of LF guided waves in the material was not considered in the modelling.

**Figure 2:**
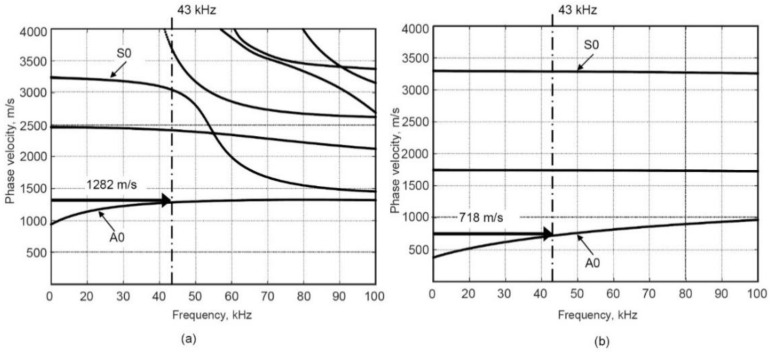
Phase velocity dispersion curves in defect-free (**a**) and defective regions (**b**) calculated by SAFE Method.

The properties of material used in SAFE analysis is presented in Table 1 [24]. GFRP plies orientations in the main spar of wind WTB and skin are +45°/−45° and 0°/90°/+45°/−45°/0°, respectively. The calculated dispersion curves in defect-free and defective regions are presented in Figure 2a,b, respectively.

It is observed that in the case of excitation of less than 50 kHz, mainly the fundamental A0 and S0 Lamb modes are propagating in both defect-free and defective regions. The frequency of interest was selected of 43 kHz according to our previous experience for LF UGWs generation in the WTB sample [24]. The phase velocities of the A0 Lamb wave modes at 43 kHz were estimated as 1282 m/s and 718 m/s in defect-free and defective regions, respectively. A significant difference in phase velocities can be effectively exploited for the detection of the internal defect.

To understand the dispersive behaviour of UGW modes and their propagation through the defects in composite materials, FE numerical modelling is an effective approach along with the experimental analysis. Numerical modelling of propagation of UGW in the WTB structure was carried out by using the Abaqus finite element software. To solve the transient wave equation, an explicit algorithm was used. To reduce the computational time, the 2D plane strain model was constructed. The frequency-dependent attenuation coefficient of low frequency guided waves in the material was not considered.

The cross-section of WTB was meshed using four-node plane strain quadrilateral elements (CPE4R) with hourglass control. The size of the finite elements was 300 µm. This size corresponds to 1/23 of the wavelength of the shortest A0 mode of the ultrasonic Lamb wave at 43 kHz frequency. Since the central difference integration method is conditionally stable, the time step Δ*t* must be smaller than the stability limit of the central difference method. In our case, the stable solutions are obtained with the time step duration of 10 ns.

Data for further analysis were collected every 100 ns and corresponds to 1/60 of the shortest period of the signal. To excite the asymmetric A0 guided wave in the WTB structure, the transient excitation force of 1 N was applied to the selected zone (Figure 1). In order to avoid reflection of the guided wave from the left side of the structure and excite the pure guided wave, all points of the left edge of the structure were used for the excitation load. The excitation force is uniformly distributed on the excitation surface. The waveform of the excitation force was 43 kHz, for three periods of sine burst with the Hamming window. Signals for analysis were collected along with the all top-surface nodes of the sample and the B-scan was constructed.

**Figure 3:**
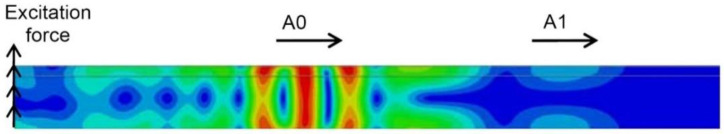
Simulated propagation of UGW in the defect-free sample by FE: the displacement field modulus at 100 µs instant of time.

**Figure 4:**
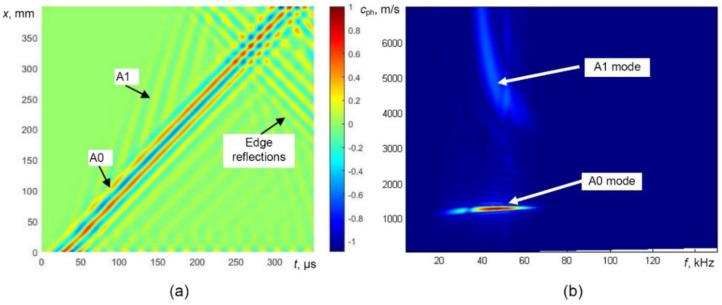
Modelling of UGW propagation in the defect-free sample: the B-scan image (**a**) and phase velocity dispersion curves reconstructed by 2D FFT (**b**).

Modulus of the displacement fields of propagating guided waves in the defect-free sample at 100 µs is presented in Figure 3. The obtained B-scan image of the normal component of the wave and 2D Fourier transform images are presented in Figure 4a,b, respectively. The propagation of the A0 mode can be easily observed at 43 kHz excitation frequency. The displacement field of the A0 mode is symmetrically distributed through the thickness of the object under investigation. The propagation of the higher-order A1 mode with higher phase velocity is also visible in Figure 3 and Figure 4a,b. After analysing the displacement field and the calculated dispersion curves with 2D FFT image (Figure 4b), it can be easily observed that the fast wave is the A1 mode. 

**Figure 5:**
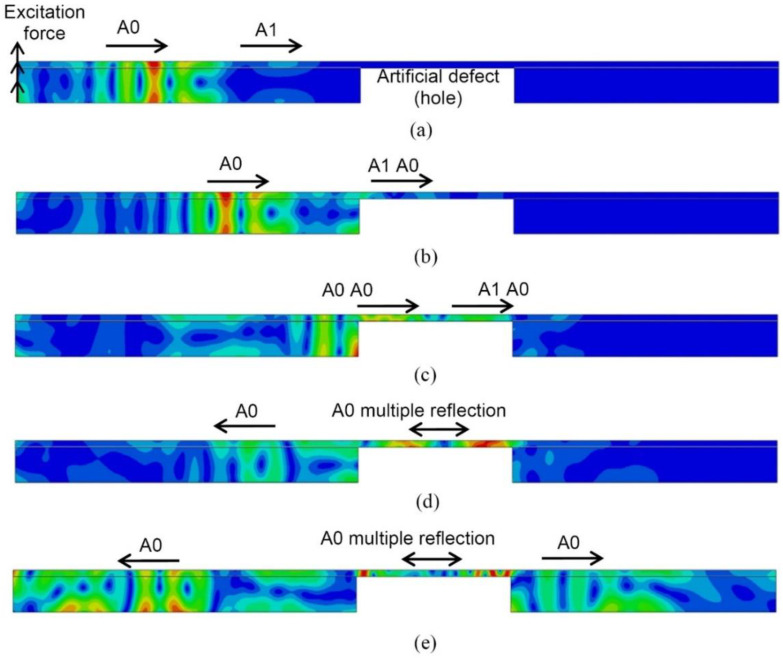
Simulated propagation of UGW in the defective sample by FE, displacement field modulus at different instants of time: 80 µs (**a**), 110 µs (**b**), 150 µs (**c**), 180 µs (**d**), 220 µs (**e**).

Furthermore, modelling has been performed for the defective WTB sample. The dynamic of wave propagation in the wind turbine blade sample with a milled-hole-type defect with a diameter of 81 mm at different time instants (from 80 µs up to 220 µs) is shown in Figure 5. It is observed that, in the case of a milled hole, strong reflection from the front edge of the defect and multiple reflections within the defective region occurs.

**Figure 6:**
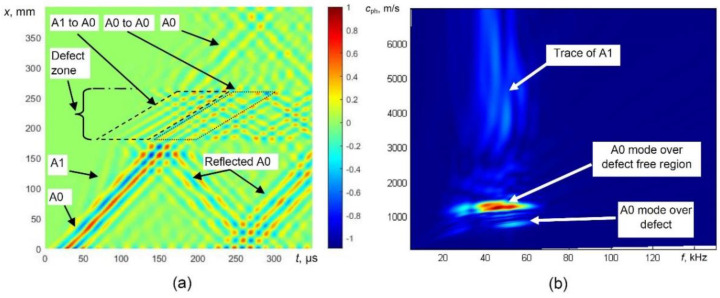
Modelling of UGW propagation in the defective sample, simulated B-scan image (**a**) and phase velocity dispersion curves reconstructed by 2D FFT (**b**).

The B-scan and 2D FFT images obtained by modelling of guided wave propagation in defective WTB are presented in Figure 6. At 43 kHz, the phase velocity of the A0 mode significantly decreases in the defective zone (Figure 6a). In this case, the conversion of the higher-order A1-guided wave mode into the A0 mode is visible as well (Figure 6a). At the back edge of the defect, the weak reflection of the A0 mode is visible and the velocity of the forward propagating A0 mode increases.

Therefore, the most reliable parameter for the indication of the presence of the defect is the variation of guided wave velocity. One of the techniques for the evaluation of phase velocity can be the 2D Fourier transform. The 2D FFT images obtained from the presented B-scan data in the case of a defect are shown in Figure 6b. The propagation of the A0 mode over the defect-free region and defective region and trace of the A1 mode is easily visible.

Due to the fact that 2D FFT is integrated over the selected time window and along the scanning axis of receiving transducer, information related to defect coordinate and particular time instant of the received signal is lost. Therefore, a suitable algorithm is required, which has been proposed in the next chapter.

## 3. The Signal Processing

**Figure 7:**
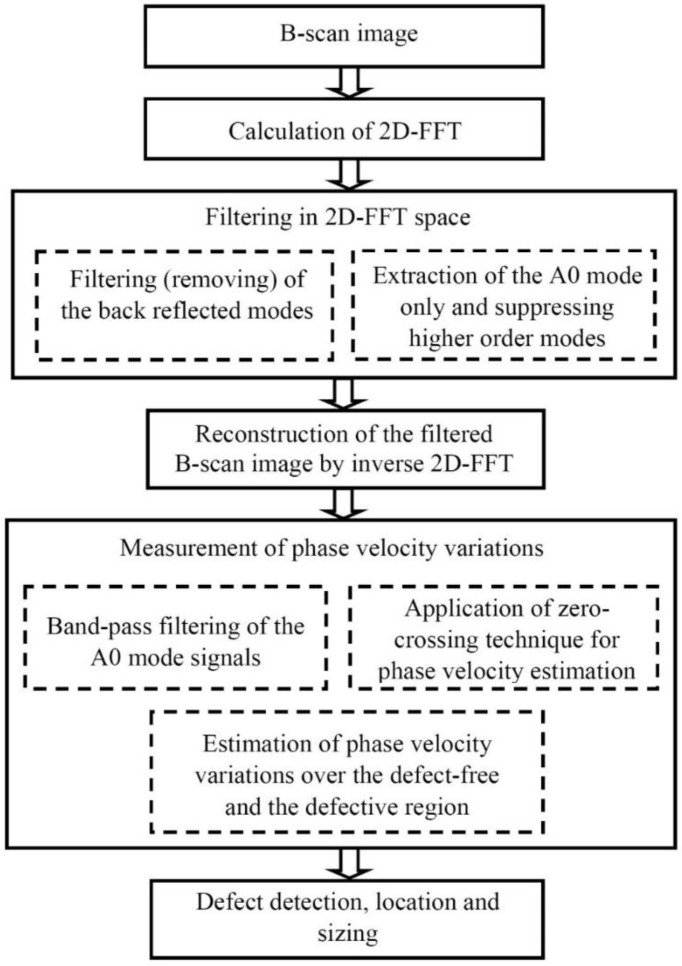
The algorithm of signal processing in frequency, spatial frequency and time domains for defect detection, location and sizing.

The flow graph depicting the proposed signal processing technique is presented Figure 7.

Firstly, the spatial filtering based on two-dimensional fast Fourier transform (2D-FFT) is performed on the ultrasonic B-scan to filter out the back-reflected signals [24,30,31]. The frequency-dependent bandpass filter with cosine-tapered windowing on the frequency-wavenumber curve in the frequency domain (after 2D FFT transform) is used as a mode separation technique to selectively extract only the A0 mode [32].

**Figure 8:**
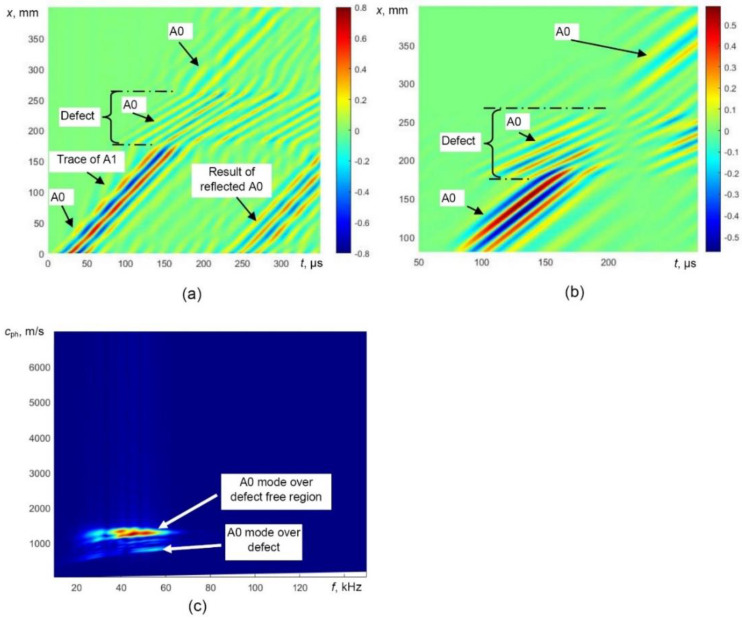
Simulated B-scan image with filtered reflected waves (**a**), selectively extracted A0 mode (**b**) and the phase velocity dispersion curves of the A0 mode reconstructed by 2D FFT (**c**).

The simulated B-scan image of the defective sample (Figure 6a) was processed by filtering out reflections from the edges of the defect and the selective extraction of the only A0 mode was performed. The processed simulated B-scan image with filtered out reflections is presented in Figure 8a. In this image, the trace of A1 is still visible. The B-scan image after removing the trace of A1 mode is presented in Figure 8b. The phase velocity dispersion curves (over defect-free and defective regions) of only A0 mode reconstructed by 2D FFT of the processed B-scans are presented in Figure 8c.

In order to estimate the size of the defect, the phase velocity variations with respect to the distance are utilized by considering two adjacent signals acquired at two different spatial measurement positions with a moving window [33]. This technique has certain advantages over the conventional 2D-FFT based phase dispersion velocity calculation [34] that do not provide the coordinate and time information.

The reconstructed B-scan image (Figure 8b) was further processed by the adapted hybrid method using the spectrum decomposition and zero-crossing techniques in order to reconstruct variation of the phase velocity along the scanning line of the sample [35,36].

The proposed hybrid method enables the reconstruction of the segments of the phase velocity dispersion curve of a Lamb wave within the bandwidth of the exciting signal. In this case, the purpose of the signal processing was to detect variations in the phase velocity of the A0 Lamb wave over defective and defect-free regions. The assumption was made that instead of reconstructing the segment of the dispersion curve, the calculation of just a single-phase velocity value at the particular spatial point could be utilized. To calculate this value, two adjacent signals *u*_x1_(*t*) and *u*_x2_(*t*) at different spatial distances *x*_1_ and *x*_2_ in the B-scan image were selected. The evaluation of the phase velocity is the key requirement; the time of flight must be measured between the same phase points of the acquired signals. Thus, the difference in time between the segments of the signals, acquired at two different spatial positions, should be smaller than half of the signal period at a particular frequency. It was determined that the minimal distance Δ*x* between these two spatial points, for registration of the A0 signals, should be shorter than half of the wavelength (at a particular frequency).

The minimal distance Δ*x* between these two spatial points for the registration of the A0 mode signals was calculated according to the above-mentioned hybrid method and can be mathematically expressed as:

(1) $$\Delta x\leq \frac{c_{{\mathrm{phA}}_{0}}}{2f_{r}},\;\;\;\Delta x\leq \frac{\lambda }{2},$$

where $c_{{\mathrm{phA}}_{0}}$ is the theoretical phase velocity of the A0 mode at *f*_r_ = 43 kHz frequency in the defect-free region, and *λ* is the wavelength of the A0 mode at frequency *f*_r_.

The basic steps of the algorithm describing the phase velocity calculation at a single point is presented below:

- The frequency spectrum of adjacent signals is calculated as:

(2) $$U_{x1}\left(f\right)=\mathrm{FT}\left[u_{x1}\left(t\right)\right],\;U_{x2}\left(f\right)=\mathrm{FT}\left[u_{x2}\left(t\right)\right],$$

where FT denotes the Fourier transform.

- The frequency spectrums are filtered by bandpass filter with predefined parameters. The filtered signals can be expressed by the following equation:

(3) $$S_{x1}\left(f\right)=U_{x1}\left(f\right)\cdot B\left(f\right),\;S_{x2}\left(f\right)=U_{x2}\left(f\right)\cdot B\left(f\right),$$

where $B\left(f\right)=e^{4\ln\left(0.5\right){\left(\frac{f-f_{c}}{\Delta B}\right)}^{2}}$ represents the frequency response of the Gaussian bandpass filter, *f*_c_ is the central frequency of the filter and Δ*B* is the filter bandwidth.

- The filtered signals are reconstructed using the inverse Fourier transform:

(4) $$s_{x1}\left(t\right)=\mathrm{IFT}\left[S_{x1}\left(f\right)\right],\;s_{x2}\left(t\right)=\mathrm{IFT}\left[S_{x2}\left(f\right)\right],$$

where IFT denotes the inverse Fourier transform.

- The envelopes *e_x_*_1_(*t*) and *e_x_*_2_(*t*) and their respective maximum values *e_x_*_1max_ and *e_x_*_2max_ of the reconstructed signals *s_x_*_1_(*t*) and *s_x_*_2_(*t*) are determined:

(5) $$e_{x1}\left(t\right)=\mathrm{HT}\left[s_{x1}\left(t\right)\right],\;e_{x2}\left(t\right)=\mathrm{HT}\left[s_{x2}\left(t\right)\right],$$

(6) $$e_{x1,\max}\left(t_{m1}\right)=max(e_{x1}\left(t\right)),\;e_{x2,\max}\left(t_{m2}\right)=max(e_{x2}\left(t\right)),$$

where HT denotes the Hilbert transform; *t_m_*_1_ and *t_m_*_2_ are the time instants at which maximum values of envelopes are calculated.

- The two zero-crossing time instants $t_{x1}^{0}$ and $t_{x2}^{0}$, which are closer to the maximum values (*e_x_*_1max_ and *e_x_*_2max_) of the envelopes, can be determined as:

(7) $$s_{x1}\left(t_{x1}^{0}\right)=0\;if\;t>t_{m1}-T_{c}$$

(8) $$s_{x2}\left(t_{x2}^{0}\right)=0\;if\;t>t_{m2}-T_{c}$$

where $T_{c}=\frac{1}{2f_{c}}$ is the half period of the signal at the central frequency *f*_c_.

- Based on the determined zero-crossing time instants, the phase velocity of the Lamb wave can be calculated as:

(9) $$c_{\mathrm{ph}}=\frac{x_{2}-x_{1}}{t_{x2}^{0}-t_{x1}^{0}}$$

**Figure 9:**
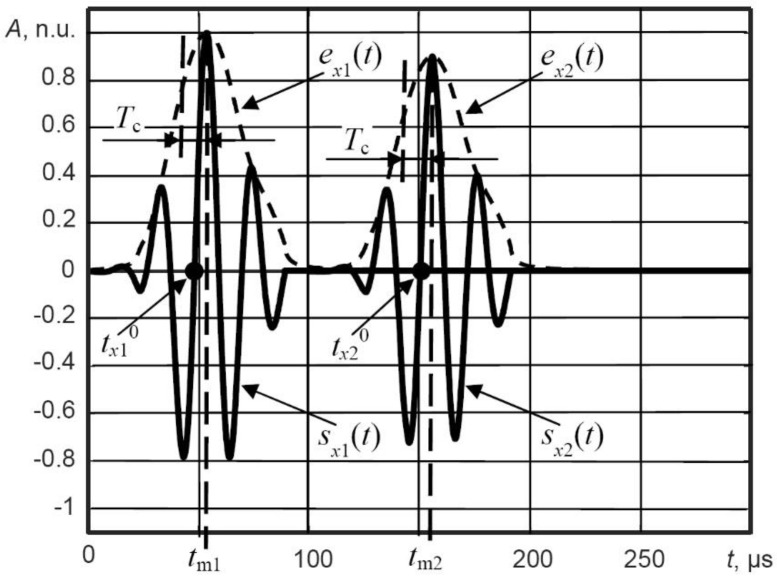
The principal demonstration for estimation of the zero-crossing time instants in the filtered signals *s_x_*_1_(*t*) and *s_x_*_2_(*t*); *A* is the normalized amplitude in normalized units (n.u.). In this demonstration, waveforms of the signals are theoretical and not to scale.

The principal demonstration for estimation of the zero-crossing time instants in the filtered signals *s_x_*_1_(*t*) and *s_x_*_2_(*t*) is presented in Figure 9. To obtain the variation in the phase velocity of Lamb wave along the scanning axis in the case of B-scan image, these calculations are repeated for each scanning point of the receiver.

In general, when using ultrasonic methods to detect a defect, the decrease in signal amplitude is one of the main criteria for determining the location of a defect. 

Generally, in ultrasonic non-destructive testing (NDT) the halving effect of the signal amplitude (at level of 0.5 or 6 dB) indicates the location of the defect. However, in the case of UGW, the signal amplitude decreases due to the effect of dispersion as a function of distance; thus, the applied conventional criterion (from ultrasonic NDT) based on the halving effect of the signal amplitude is not appropriate. Therefore, a new criterion based on the measurement of the phase velocity variation along the scanning line of the receiving transducer is developed and presented. So, the main questions are how to measure the phase velocity variation and on what basis to perform such measurement. 

The phase velocity threshold $c_{\mathrm{ph},\mathrm{thr}}$, based on the intermediate value of the estimated phase velocity variation between the maximum and minimum values is proposed to be used as a new criterion for the defect evaluation. It does not require calibration and does not depend on the sample under investigation. In addition, using this new defect detection criterion, the coordinates of the defect can be estimated. It can be mathematically expressed by the following equation:

(10) $$c_{\mathrm{ph},\mathrm{thr}}=\min\left(c_{\mathrm{ph}}\left(x\right)\right)+0.5\cdot \Delta c_{\mathrm{ph}},\;\;\;\Delta c_{\mathrm{ph}}=\max\left(c_{\mathrm{ph}}\left(x\right)\right)-\min\left(c_{\mathrm{ph}}\left(x\right)\right)$$

**Figure 10:**
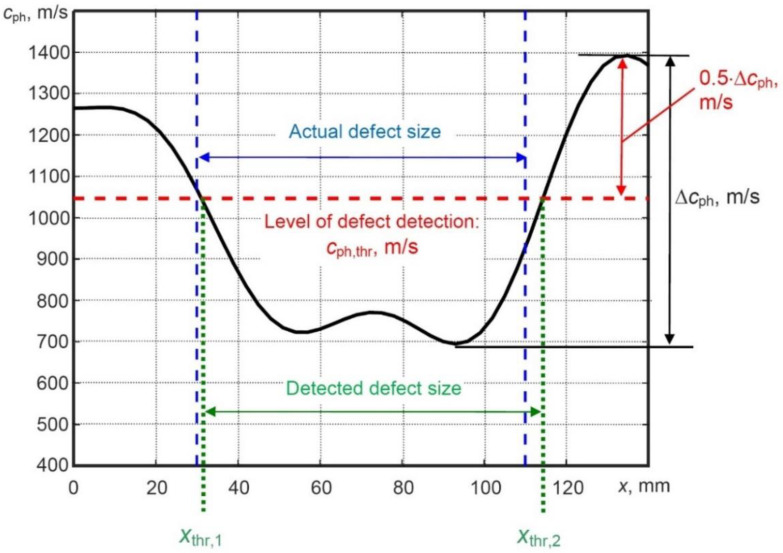
Phase velocity variation of the A0 Lamb wave for the defect detection and estimation of its location and size in the case of simulated B-scan image.

As mentioned above, a halved signal amplitude in NDT usually indicates the location of the defect (e.g., level of 0.5 or −6 dB), therefore, the multiplier coefficient of 0.5 is used for the phase velocity threshold calculation. The application of the proposed phase velocity variations technique based on a new criterion for detection, location and sizing of the defect in the case of the simulated model is shown in Figure 10. 

The actual size of the defect is 81 mm; *x* coordinates of the first and second edges of the defect are *x*_1_ = 30 mm and *x*_2_ = 111 mm, respectively. The minimum and maximum phase velocities were obtained as 700 m/s and 1400 m/s, respectively. Using the proposed measurement method, the obtained value of $c_{\mathrm{ph},\mathrm{thr}}$ was 1050 m/s. In order to locate the defect, *x* coordinates of the first ($x_{\mathrm{thr},1}$) and second ($x_{\mathrm{thr},2}$) edges of the defect were estimated. The estimated coordinate $x_{\mathrm{thr},1}$ of the first edge of the defect was 31.1 mm with a relative error of 3.7%. On the other hand, the estimated coordinate $x_{\mathrm{thr},2}$ of the second edge of the defect was 114.3 mm with a relative error of 3%. Detection of the defect size was performed by the difference of *x* coordinates ($x_{\mathrm{thr},2}-x_{\mathrm{thr},1})$. The obtained value of defect size was 83.2 mm with a relative error of 2.7%. 

## 4. The Experimental Investigation

**Figure 11:**
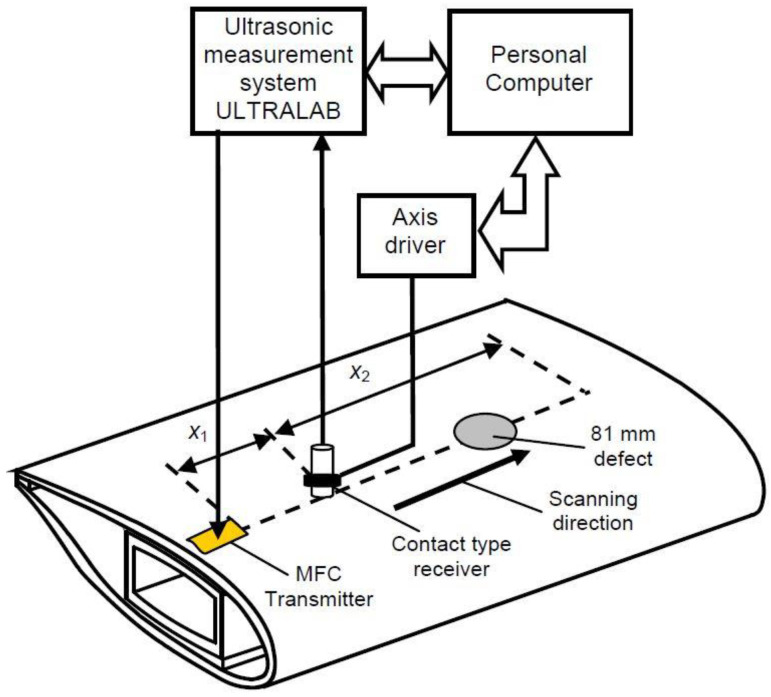
Experimental set-up of WTB inspection.

The experimental validation of results obtained from the modelling is also performed by using the low-frequency (LF) ultrasonic system developed by the Ultrasound Research Institute of Kaunas University of Technology [2,37]. The WTB segment was constructed from glass fibre reinforced plastic (GFRP) material. The macro-fibre composite (MFC) transducer of P1-type (M-2814-P1) is used to excite the guided waves at 43 kHz, whereas the contact-type piezoceramic wideband transducer is used as a receiver. The experimental setup of WTB inspection is presented in Figure 11. Each wave mode (symmetric or asymmetric) has a different wavelength, velocity, or wave patterns across the thickness of the structure and these variations significantly depend on the frequency of excitation along with the material properties. However, in our previous research [38], we observed that the resonant frequency of the MFC transducer was 43 kHz. That is why the 43 kHz frequency was selected as the excitation frequency in this work and the transducer was excited by a rectangular pulse having a duration of a half (11.6 µs) of the single period (23.25 µs). The wideband contact-type ultrasonic receiving transducer was scanned up to the distance of *x*_2_ = 160 mm away from the transmitter with a step of 0.1 mm. The initial distance between the transmitter and receiver was *x*_1_ = 30 mm.

**Figure 12:**
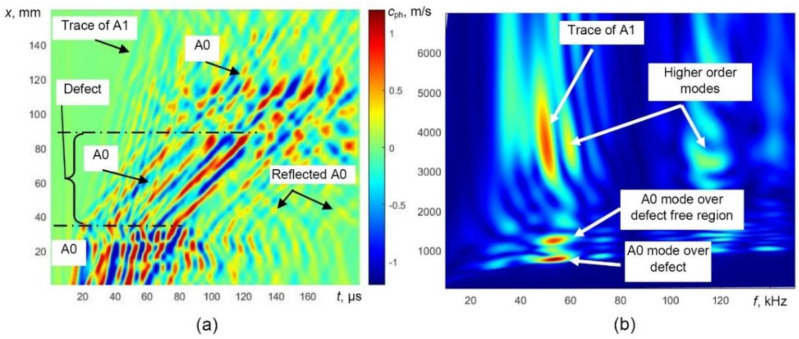
Propagation of UGW in the defective sample and measured B-scan image (**a**) and phase velocity dispersion curves reconstructed by 2D FFT (**b**).

The obtained B-scan image, propagation effects of UGW in defective WTB sample, and the visible interaction effects of UGW with the defect are presented in Figure 12a. It is possible to observe the reflections of the A0 mode from the edges of the defect and also the trace of the A1 mode. Phase velocity dispersion curves reconstructed by 2D FFT are presented in Figure 12b. It should be noted that the wave energy of the A1 mode was much weaker than the A0 modes in the FE results (Figure 6b); however, the A1 mode shows much stronger wave energy in the experimental result (Figure 12b). This can be explained by applying different guided waves’ excitation forces in numerical modelling and experimental investigations. In the case of numerical modelling, the excitation force was applied in such a way that the A0 mode would be excited as cleanly and efficiently as possible due to excitation force perpendicular to the surface of the sample. Even in this case, the additional weak A1 was excited. During the experiment, the MFC transducer was used to excite the ultrasonic guided waves, possessing *out of plane* and *in plane* displacement components of guided waves. Therefore, the A1 mode was excited much more strongly. In real experimental conditions, the slight difference in results may also appear due to the sensitivity of transducers, environmental factors, the variable thickness of WTB and the condition of the object as well.

**Figure 13:**
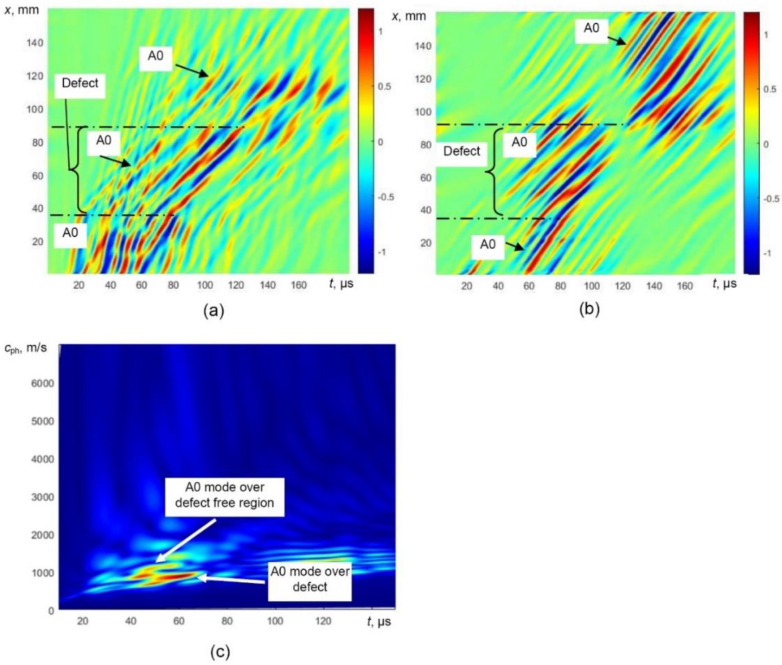
Processed B-scan image (**a**), extracted A0 mode (**b**) and the phase velocity dispersion curves of the A0 mode reconstructed by 2D FFT (**c**).

The obtained B-scan image was processed using the algorithm presented in a previous chapter. The processed B-scan images after filtering out the reflections and selectively extracted A0 mode are presented in Figure 13a,b. The phase velocity dispersion curves of the A0 mode, over the defect-free and defective regions, reconstructed by 2D FFT are presented in Figure 13c.

**Figure 14:**
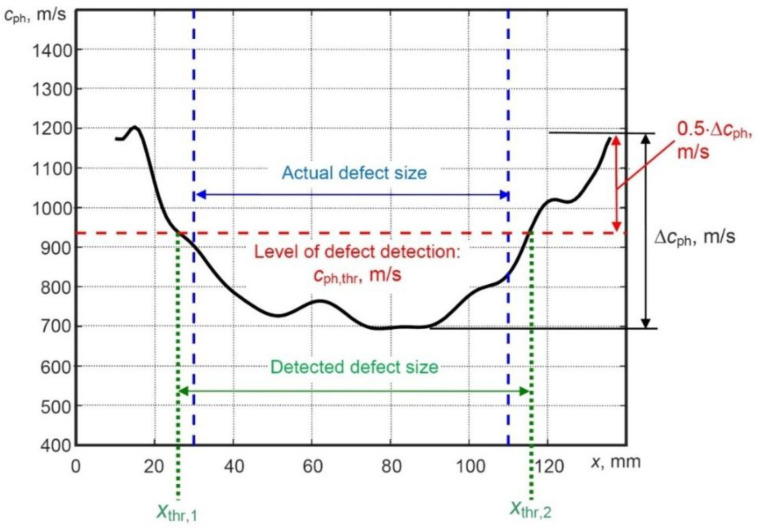
Phase velocity variation of the A0 Lamb wave for the defect detection and estimation of its location and size in the case of simulated B-scan image.

Furthermore, analysis of phase velocity variations for the A0 mode along the scanning line of the WTB sample was performed in the case of the experimental B-scan image of selectively extracted A0 mode (Figure 13c). Then, the proposed method for measurement of phase velocity variations was applied. The minimum and maximum phase velocity (*c*_ph_) was observed as 700 m/s and 1200 m/s, respectively. For defect detection, the threshold criteria (*c*_ph,thr_) described in Section 3 was used and the obtained value was $c_{\mathrm{ph},\mathrm{thr}}=$ 950 m/s. Phase velocity variations and defect analysis is shown in Figure 14.

For defect location, *x* coordinates of first ($x_{\mathrm{thr},1}$) and second ($x_{\mathrm{thr},2}$) edges of the defect were estimated (Figure 14). The estimated coordinate $x_{\mathrm{thr},1}$ of the first edge of the defect was 26.25 mm with a relative error of 12.5%. The estimated coordinate $x_{\mathrm{thr},2}$ of the second edge of the defect was 115.34 mm with a relative error of 3.9%. The size of the defect is estimated by the difference between these two parameters ($x_{\mathrm{thr},2}-x_{\mathrm{thr},1}$). The size of defect was estimated as 89.1 mm with a relative error of 10%.

## 5. Discussion and Conclusions

In this research, an effective technique based on phase velocity variations of UGW has been developed and proposed for defect detection, location and sizing. The artificially milled defect having a diameter of 81 mm on the segment of WTB is used for the investigation. The signal processing method based on two adjacent signals acquired at two different spatial measurement positions with a moving window is used to obtain the phase velocity variations with respect to the scanning distance along the sample of WTB. In this way, the variations in the phase velocity of the A0 Lamb wave over defective and defect-free regions are obtained. To measure the phase velocity variations, the threshold, based on the intermediate value between the maximum and minimum values, is proposed and used as a new criterion for the defect evaluation. In this way, not only the defect detection and location are being estimated, but the edge coordinates that indicate the size of the defect as well.

The proposed technique was verified using simulation and experimental techniques. By using the proposed technique for analysis of the simulated B-scan image, the size of an 81-mm defect was obtained as 83.2 mm. The first and second coordinates of defect edges were estimated with relative errors of 3.7% and 3%, respectively. The calculation of the relative error showed that using the proposed technique in the case of modelling, the defect size can be estimated at 2.7%. In the case of an experimentally measured B-scan image, the size of defect was estimated as 89.1 mm with a relative error of 10%. The first and second coordinates of defect edges have been estimated with relative errors of 12.5% and 3.9%, respectively. The obtained results prove that the proposed new criterion based on the phase velocity variations could be used for the defect parameterization and quality control of composite-based complex engineering constructions.

Further investigations are in progress in order to determine the advantages and limitations for the applicability of the proposed technique.

## Figures and Tables

**Table 1:** GFRP material properties used in modelling.

**Parameters**	**Numerical Value**
**Paint (Surface layer):**	
Density (*ρ*)	1270 kg/m^3^
Young’s modulus (E)	4.2 GPa
Poisson’s ratio (υ)	0.35
**Unidirectional GFRP Layer:**	
Density (*ρ*)	1828 kg/m^3^
Young’s modulus (E_1_)	42.5 GPa
Young’s modulus (E_2_)	10 GPa
Poisson’s ratio (υ_12_)	0.26
Poisson’s ratio (υ_23_)	0.4
In-plane shear modulus (G_12_)	4.3 GPa
**Epoxy:**	
Density (*ρ*)	1260 kg/m^3^
Young’s modulus (E)	3.6 GPa
Poisson’s ratio (υ)	0.35

## Data Availability

Not applicable.
